# Concurrent presence of inflammation and obstructive sleep apnea exacerbates the risk of metabolic syndrome: A KoGES 6-year follow-up study

**DOI:** 10.1097/MD.0000000000004488

**Published:** 2017-02-17

**Authors:** Jinkwan Kim, Dae Wui Yoon, Seung Ku Lee, Seunggwan Lee, Kyung-Mee Choi, Thomas J. Robert, Chol Shin

**Affiliations:** a Department of Biomedical Laboratory Science, College of Health Science, Jungwon University, Geo-San; b Institute of Human Genomic Study, Korea University Ansan Hospital, Korea University, Ansan; c Department of Health and Integrative Science, College of Health Science, Korea University, Seoul, Republic of Korea; d Division of Pulmonary, Critical Care and Sleep Medicine, Department of Medicine, Beth Israel Deaconess Medical Center, Boston, MA; e Department of Pulmonary Sleep and Critical Care Medicine Disorder Center, College of Medicine, Korea University, Ansan, Republic of Korea.

**Keywords:** cardiovascular disease, high-sensitivity C-reactive protein, inflammation, metabolic syndrome, obstructive sleep apnea

## Abstract

Obstructive sleep apnea (OSA) leads to multiple end-organ morbidities that are mediated by the cumulative burden of oxidative stress and inflammation. Both OSA and inflammation play key roles in increased risk of cardiovascular disease (CVD). Thus, we hypothesized that the combination of inflammation and OSA could accelerate the development of metabolic syndrome (MetS) in a large cohort study.

A total of 1835 participants were randomly selected from the ongoing Korean Genome and Epidemiology Study for the years between 2007 and 2015. Overnight polysomnography was performed on each participant. Blood was drawn for biochemical analyses. Participants with high or low inflammation were divided by high-sensitivity C-reactive protein (hsCRP). MetS was defined using the criteria of the modified National Cholesterol Education Program, Adult Treatment Panel III.

The prevalence of MetS was higher among the subjects with OSA and high hsCRP levels than among the other corresponding groups. The incidence of MetS among the 4 groups stratified by OSA and inflammation status at the 6-year follow-up was 11.8%, 19.9%, 25.8%, and 36.0% (HsCRP[−]/OSA[−] vs HsCRP[+]/OSA[−] vs HsCRP[−]/OSA[+] vs HsCRP[+]/OSA[+], *P* < 0.01). After adjusting for age, sex, smoking, alcohol status, BMI, and change in BMI (*Δ*BMI) in a multiple logistic regression, the subjects with OSA and high hsCRP levels at follow-up had a 2.22-fold risk of developing MetS, as compared with those with no-OSA and low hsCRP levels (*P* < 0.01).

MetS is more prevalent in the concurrent presence of inflammation and OSA. The combination of these conditions is associated with higher risk of MetS. Additional research is needed to help further define the significance of the combined effect of OSA and subclinical inflammation on the development of MetS in the context of reduction of CVD risk.

## 1 Introduction

Obstructive sleep apnea (OSA) is characterized by repeated events of partial or complete upper airway obstruction during sleep, leading to hypoxemia, and sleep fragmentation. In the last several decades, increasing obesity rate has led to a remarkable increase in the prevalence of OSA and has gained an increasing scientific attention, given the prominent cardiovascular and metabolic morbidities associated with this condition.^[1–7]^ Though the underlying mechanisms that OSA leads to metabolic abnormalities are not fully understood, systemic inflammation has emerged as a key contributor to the occurrence and magnitude of OSA-associated morbidity.^[7–9]^ High-sensitivity C-reactive protein (HsCRP) is a robust biomarker of underlying systemic inflammation and appears to be an important marker in both cardiovascular disease and metabolic syndrome.^[10,11]^ A number of studies have reported that elevated inflammatory markers such as TNF-α, IL-6, and hsCRP were found among OSA patients, and these markers were positively correlated with excessive daytime sleepiness.^[3,9,12]^ However, not all of OSA patients exhibited increased systemic inflammation,^[13–15]^ suggesting that an interaction effect between genetic variances and various environmental factors might constitute an important determinant of an inflammation phenotype in OSA.^[15–18]^ Interestingly, a recent study also suggests that epigenetic modification could account for the discrepancy of an inflammatory phenotype in OSA.^[19,20]^

Metabolic syndrome (MetS) includes a constellation of metabolic derangements, such as glucose intolerance, central obesity, hypertension, and dyslipidemia, all well-known risk factors for cardiovascular disease (CVD). A growing body of evidence suggests that OSA is associated with a number of metabolic alterations.^[11,21]^ Although large cross-sectional studies have shown that OSA can be associated with impaired glucose tolerance and insulin sensitivity, prospective studies have produced conflicting results, owing to methodological limitations such as small sample sizes, subjective measurement of OSA, or inadequate consideration of various confounding factors.^[11,21,22]^ Moreover, no study has been published regarding whether concurrent presence of inflammation and OSA increases the risk of MetS and exacerbates the development of MetS in a prospective population-based study. Therefore, the purpose of the present study was to investigate whether the presence of inflammation among those with OSA would accelerate the risk of MetS in a prospective cohort study.

## 2 Subjects and methods

### 2.1 Subjects

Participants in the present study were part of a larger study, the Korean Genome and Epidemiology Study (KoGES), which is an ongoing, population-based cohort study that started in 2001 under the original title, the Korean Health and Genome Study. Detailed information on the study design and aims of the KoGES has been previously reported.^[23–25]^ In brief, the original study was designed to establish a representative adult cohort in the city of Ansan, Korea, and to identify the epidemiologic characteristics and the frequency and determinants of chronic diseases among Koreans. From June 2001 to January 2003, a longitudinal cohort was formed, consisting of 5015 participants (2521 men and 2494 women ages 40–69 years) who participated in a comprehensive health examination and on-site interviews at Korea University Ansan Hospital. Follow-up assessments were conducted biennially with scheduled site visits. At each visit, participants signed an informed consent form, which was approved by the Human Subjects Review Committee at Korea University Ansan Hospital. Data from the 4th biennial examination from March 2007 to February 2009 and the 7th examination from March 2013 to February 2015 (follow-up) were used in the current study. Polysomnography (PSG) was included in the study protocol in September 2009 in about half of the KoGES participants. Although PSG will eventually be administered to the entire study population, subjects for the present study include only those with PSG data acquired between September 2009 and February 2012. After excluding participants who had missing data and those with extreme outliers of biochemical data, a total of 1835 individuals (968 men and 867 women) were recruited into the current study. Participants were also excluded if they had known any defined systemic inflammatory disease, genetic abnormality, or received any treatment for OSA at baseline and follow-up. For the purpose of the present study, participants were divided into 4 groups based on hsCRP level among those with OSA and those without (non-OSA).

### 2.2 Overnight polysomnography

An overnight PSG was performed at each participant's home using a portable device (Embletta X-100; Embla Systems, San Carlos, CA), as previously described.^[26]^ The recording channels were as follows: 1 electroencephalography (C4-A1), 1 electrooculography, 1 chin electromyography, 1 modified lead II electrocardiography, 1 airflow from nasal airflow pressure transducer, 2 respiratory efforts from chest and abdominal respiratory inductance plethysmography, 1 pulse oximeter, and 1 position sensor. Obstructive sleep apnea was defined when airflow dropped to ≥90% of baseline with ongoing chest and abdominal movement, and hypopnea as a reduction in airflow by ≥30%, associated with at least 4% oxygen desaturation. The duration threshold for these respiratory events was 10 seconds. The apnea-hypopnea index (AHI) was calculated. OSA was defined if the AHI score was greater than 10. Arousals were defined according to the American Academy of Sleep Medicine Scoring Manual.^[27,28]^

### 2.3 Anthropometric and biochemical data

All the study participants provided information about personal health history, sleep habits, and lifestyle. Blood pressure was measured in a standardized manner by a trained research assistant using a mercury sphygmomanometer. At least 2 blood pressure readings were recorded at 30-second intervals, and the average value was used as a measure of systolic and diastolic blood pressure. BMI was calculated as weight in kilograms divided by height in meters squared. Waist circumference was measured at the midpoint between the lower rib margin and the iliac crest in a standing position. Daytime sleepiness was assessed with the Epworth Sleepiness Scale (ESS). Blood was drawn for biochemical analysis after overnight fasting. Plasma glucose, serum triglycerides, high-density lipoprotein (HDL) cholesterol, and hsCRP levels were measured with an autoanalyzer (ADVIA 1650 and 1800, Siemens, Tarrytown, NY). Insulin level was measured with an immunoradiometric assay kit (INS-IRMA kit; BioSource, Nivelles, Belgium). High hsCRP level was defined as >1.46 mg/dL for male and >1.16 mg/dL for female, corresponding to the 75th percentile among non-OSA controls.

### 2.4 Metabolic syndrome

Data were also collected on factors used to define components of MetS. Based on the Third Report of the National Cholesterol Education Program Expert Panel on Detection, Evaluation, and Treatment of High Blood Cholesterol in Adults (NCEP-III),^[29]^ the components included abdominal obesity, low levels of HDL-cholesterol, hypertension, hypertriglyceridemia, and high fasting glucose. High blood pressure was defined when systolic blood pressure was 130 mm Hg or higher, diastolic blood pressure was 85 mm Hg or higher, or taking antihypertensive medication. Low HDL-cholesterol was defined as the level lower than 40 mg/dL for male and 50 mg/dL for female. Hypertriglyceridemia was defined with the concentration level of 150 mg/dL or greater. High fasting glucose was defined as the glucose level of at least 100 mg/dL or when a subject was receiving oral hypoglycemic agents or insulin therapy. Abdominal obesity was defined as a waist circumference wider than 90 cm in male and 85 cm in female.^[30]^ Each of the participants was assigned a metabolic score calculated by adding the number of positive diagnostic criteria by the NCEP-III. MetS was defined if a participant received a score of 3 or greater out of 5. The index of homeostasis model assessment insulin resistance (HOMA-IR) was calculated as fasting glucose (mmol/L) × fasting insulin (μ U/mL)/22.5. Obesity was defined with BMI ≥25 kg/m^2^, according to the Asian-specific BMI cutoff from the World Health Organization Report.^[31]^ The incidence of MetS was defined as the percentage of participants who were newly diagnosed with MetS during the 6-year follow-up period among those without MetS at baseline.

### 2.5 Statistical analysis

Statistical mean difference was examined for normal variates by using ANOVA, and probabilistic distribution was compared for non-normal variates by using the Kolmogorov–Smirnov test. Multivariate logistic regression was applied, with adjusting for the factors identified to be significant risk factors for MetS in univariate analyses, which were age, sex, smoking status, alcohol use, baseline BMI, and change in BMI at the 6-year follow-up (*Δ*BMI). Bonferroni correction was applied for pairwise comparisons. Adjusted odds ratios were estimated with 95% confidence intervals (CIs), referencing to that for the participants with neither inflammation nor OSA. Statistical significance was identified at the 0.05 significance level. All the statistical analyses were done using SPSS (IBM SPSS Statistics 20.0).

## 3 Results

### 3.1 Study population

In the present study, we conducted a prospective longitudinal study of 1835 participants derived from the Korean Genome and Epidemiology Study initiated in 2001. Participants underwent baseline PSG and continued to participate for 5.89 years on average in the examination on whether the concurrent presence of inflammation with OSA may accelerate the risk of MetS development. Among the 1835 participants at baseline examination, 212 subjects (115 males and 97 females) were dropped out over the 6-year follow-up period (nonresponse rate, 11.5%). There were no significant differences between the respondents and nonrespondents in the general characteristics including sex, smoking status, alcohol use and BMI, and PSG examination for AHI, respectively (*P*>0.05). However, mean age was older for nonrespondents (57.5±8.5 years old) than for respondents (55.5±7.0 years old), and the difference was statistically significant (*P* < 0.05). In Table 1, demographic, polysomnographic, and biochemical characteristics are presented for each of the 4 groups classified by OSA and hsCRP levels at baseline. At baseline, participant's mean age was 55.5 years old, and 52.8% was males. There was no significant difference in average length of follow-up years among the 4 groups (HsCRP[−]/OSA[−] vs HsCRP[+]/OSA[−] vs HsCRP[−]/OSA[+] vs HsCRP[+]/OSA[+], 5.89 ± 0.23 years vs 5.87 ± 0.22 years vs 5.91 ± 0.20 years vs 5.91 ± 0.21 years, *P* = 0.29). PSG data for AHI and SaO2 nadir showed a significant group difference, respectively (*P* < 0.01). The hsCRP levels of participants with low and high hsCRP levels among non-OSA and OSA participants were 0.52, 2.72, 0.64, and 2.97 at baseline and 0.97, 1.84, 1.00, and 2.55 at follow-up, respectively. Metabolic profiles, including HOMA-IR, glucose, triglyceride, and HDL cholesterol levels, were significantly different among the 4 groups (*P* < 0.01); however, total cholesterol did not significantly vary. For the corresponding 4 groups, the metabolic score calculated by summing the number of positive diagnostic criteria was 1.30, 1.78, 1.88, and 2.23, respectively, at baseline, and 1.61, 2.04, 2.18, and 2.69, respectively, at the 6-year follow-up.

**Table 1:**
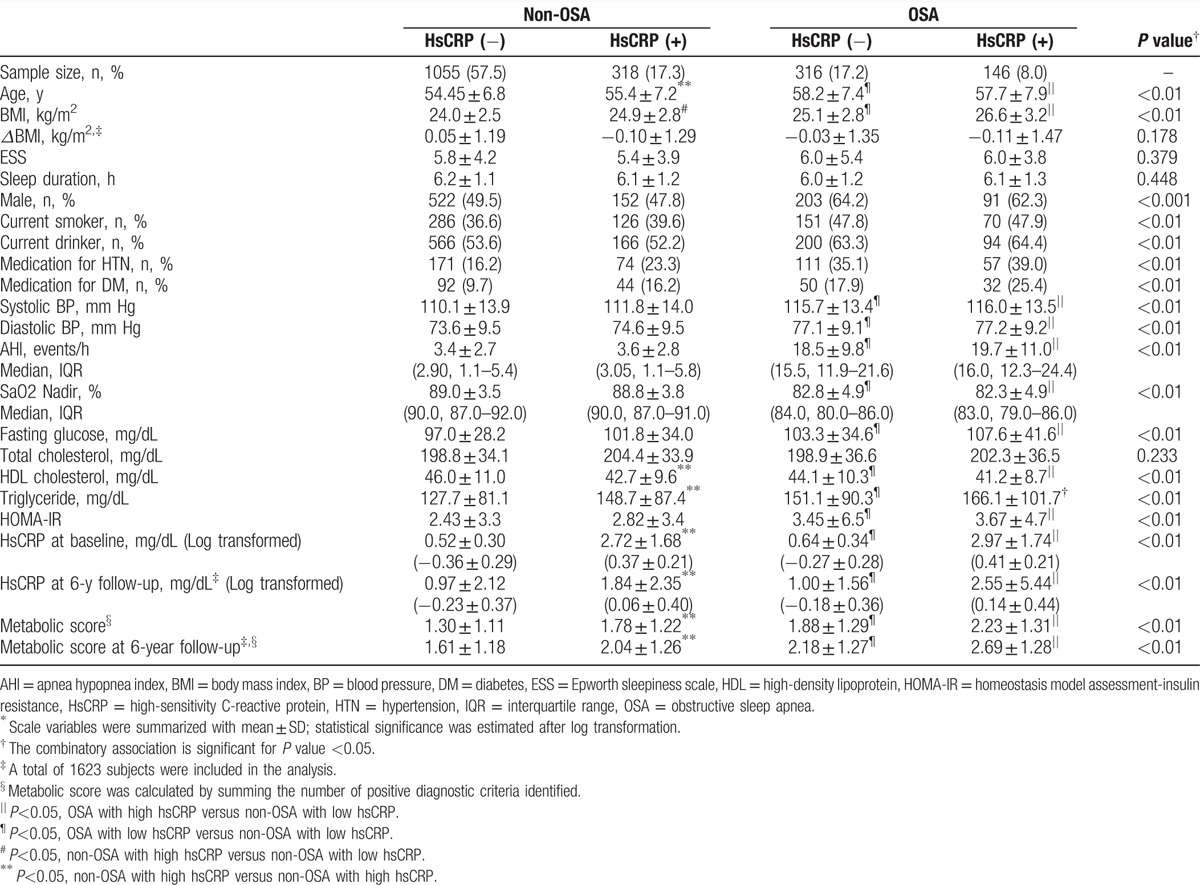
General characteristics of study participants according to the presence of inflammation and OSA^∗^.

### 3.2 Prevalence and incidence of metabolic syndrome according to inflammation and OSA

The percentages of MetS components, including abdominal obesity, hypertriglyceridemia, low HDL cholesterol, hypertension, and high fasting glucose, are shown in Table 2. Among the 4 groups, the prevalence of each component of MetS except hypertension was the highest particularly among the participants with high hsCRP level and OSA. Similarly, the prevalence of MetS diagnosed by the modified NCEP- III criteria significantly increased across the 4 groups as also seen in Table 2 (HsCRP[−]/OSA[−] vs HsCRP[+]/OSA[−] vs HsCRP[−]/OSA[+] vs HsCRP[+]/OSA[+], 15.1% vs 26.4% vs 30.7% vs 41.8%, respectively; *P* < 0.001).

**Table 2:**
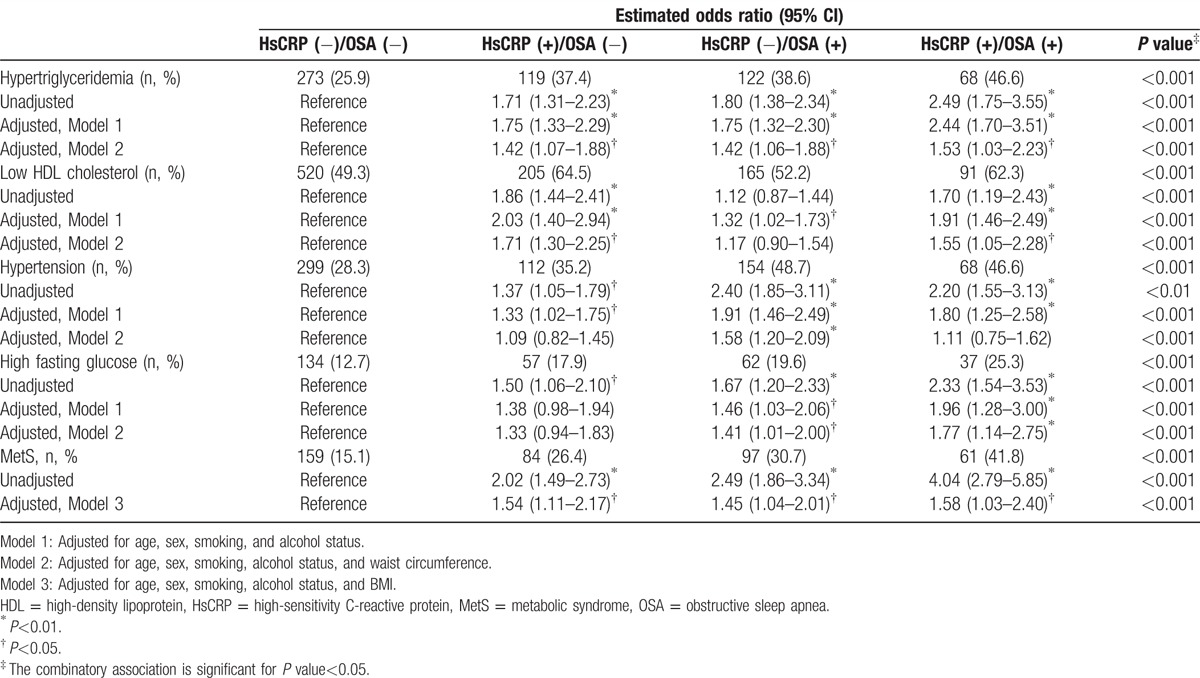
Estimated odds ratios for the risk of MetS according to the combinatory presence and absence of inflammation and OSA at baseline.

**Figure 1:**
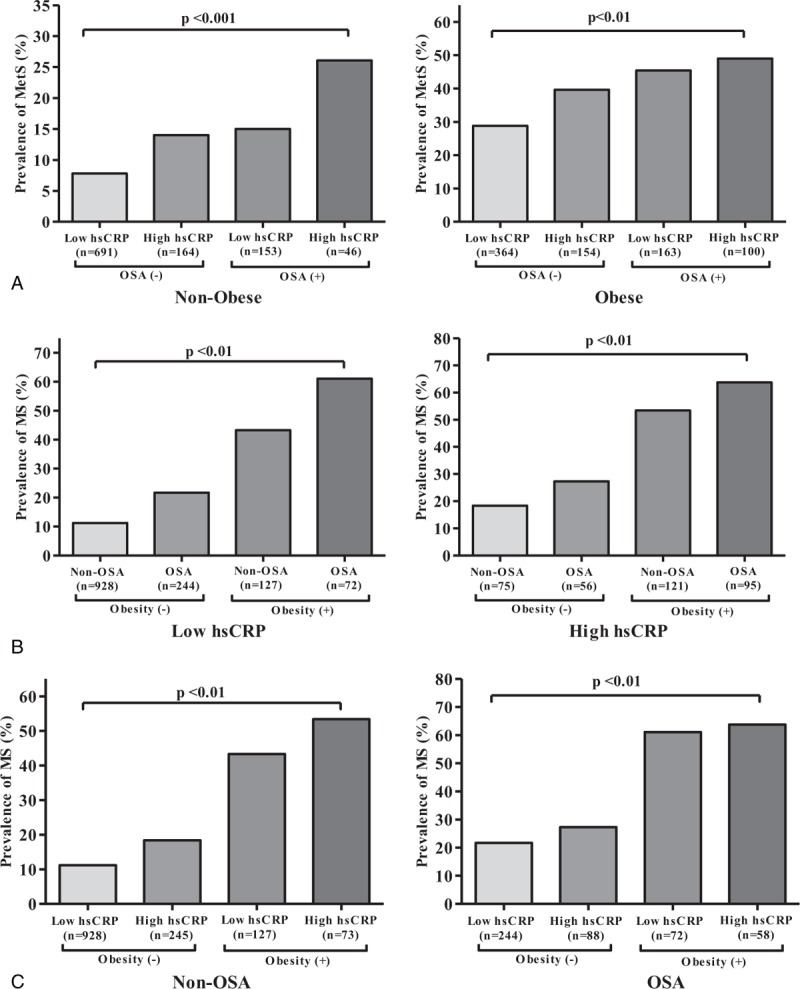
Prevalence of MetS among the concurrent status of 4 groups marginally stratified by (A) obesity, (B) inflammation, and (C) OSA, respectively. Obesity was defined with BMI ≥25 kg/m^2^ according to the Asian-specific BMI cutoffs from the World Health Organization Report. MetS = metabolic syndrome, OSA = obstructive sleep apnea.

**Figure 2:**
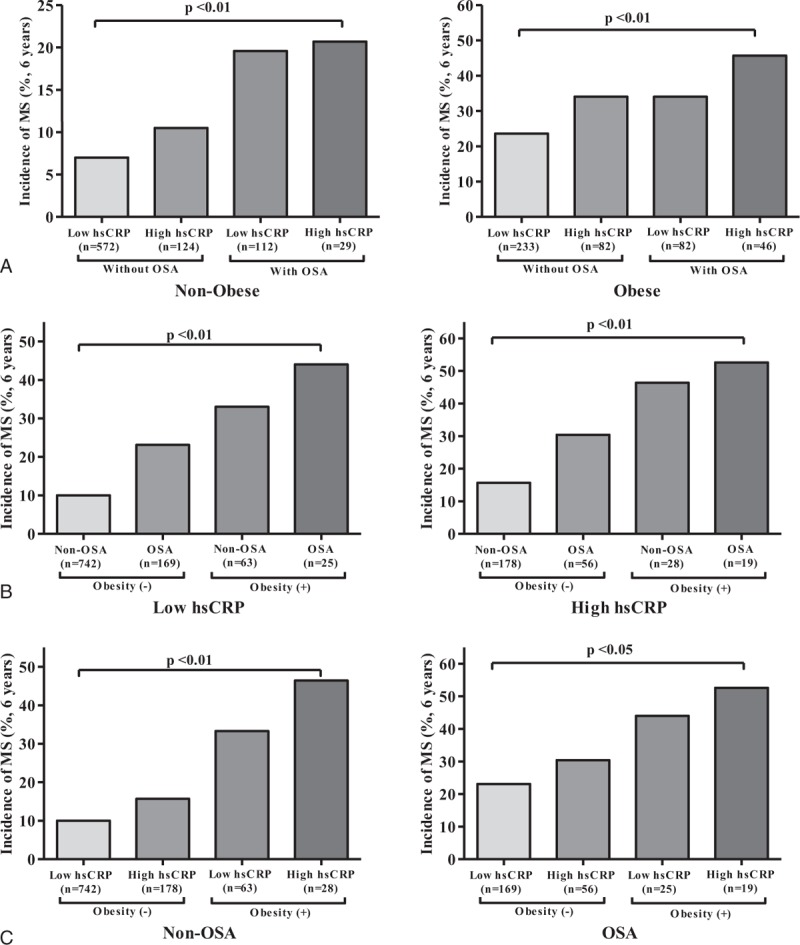
Incidence of MetS among the concurrent status of 4 groups marginally stratified by (A) obesity, (B) inflammation, and (C) OSA, respectively, at 6-year follow-up. An incidence was defined as the percentage of participants who were newly diagnosed with MetS during the 6 years of follow-up period among those without MetS at baseline (n = 1280). MetS = metabolic syndrome, OSA = obstructive sleep apnea.

Because obesity is expected to contribute to increase MetS rate as a confounding factor, we compared the prevalence of MetS in nonobese to obese participants among the 4 combination groups. Even after adjusting for obesity (BMI≥25 kg/m^2^), the prevalence of MetS among the 4 groups was significantly different as follows (see also Fig. 1A): HsCRP[−]/OSA[−] vs HsCRP[+]/OSA[−] vs HsCRP[−]/OSA[+] vs HsCRP[+]/OSA[+], 7.8% vs 14.0% vs 15.0% vs 26.1% among the nonobese participants (*P* < 0.001), and 28.8% vs 39.6% vs 45.4% vs 49.0% among the obese participants (*P* < 0.01). Plots in Fig. 1A have different upper limits with a half-smaller unit for nonobese than for obese. Using *χ*^2^ test, the association between the prevalence of MetS and hsCRP was identified not to be significant not only for nonobese but also obese, respectively (*P*> 0.05). Moreover, there was a significant difference in the incidence of MetS at the 6-year follow-up among the 4 combination groups in the nonobese and obese participants, respectively (see Fig. 2A): HsCRP[−]/OSA[−] vs HsCRP[+]/OSA[−] vsHsCRP[−]/OSA[+] vs HsCRP[+]/OSA[+], 7.0% vs 10.5% vs 19.6% vs 20.7% among the nonobese participants (*P* < 0.01), and 23.6% vs 34.1% vs 34.1% vs 45.7% among the obese participants (*P* < 0.01).

Additionally, adjusting for inflammation (Fig. 1B), baseline prevalence for MetS was significantly different among the another 4 combination groups corresponding to the adjustment, that is, Obesity[−]/OSA[−] vs Obesity[−]/OSA[+] vs Obesity[+]/OSA[−] vs Obesity[+]/OSA[+] (*P* < 0.01). It was consistent for each of the subgroups for inflammation that the highest prevalence of MetS was in the group of Obesity[+]/OSA[+] followed by Obesity[+]/OSA[−] and Obesity[−]/OSA[+], and the least prevalence was in the group of Obesity[-]/OSA[−]. In Fig. 1B for obesity (+), there was a significant association between MetS and OSA presence for only in those with low hsCRP, and not for those with high hsCRP (*P* values 0.002 and 0.159, respectively). This indicated a significant interactive association between MetS and OSA presence in those with low hsCRP only. Similarly, we adjusted for OSA (Fig. 1C), and compared the baseline prevalence of MetS among the corresponding another 4 subgroups, that is, hsCRP[−]/Obesity[−] vs hsCRP[+]/Obesity[−] vs hsCRP[−]/Obesity[+] vs hsCRP[+]/Obesity[+] in each of the subgroups for OSA. It was consistent in the subgroups for OSA that the highest prevalence of baseline MetS was in the group of hsCRP[+]/Obesity[+], followed by hsCRP[−]/Obesity[+] and hsCRP[+]/Obesity[−], and the least in the group of hsCRP[−]/Obesity[−]. In Fig. 1C for obesity (+), an association of MetS with hsCRP was not significant in the presence of OSA (*P* value 0.093), but significant in its absence, that is, non-OSA (*P* value 0.001). In those without OSA, the risk of MetS was 1.8 times (95% CI, 1.1–3.1) higher for high hsCRP than for low hsCRP. Thus, this also indicated that there was a significant interactive association between MetS and hsCRP, but only in those without OSA.

Similarly, the 6-year follow-up incidence rate for MetS was compared with adjusting for inflammation (Fig. 2B), and adjusting for OSA (Fig. 2C), respectively. As seen in the figures, there was a consistent trend to increase in the 6-year follow-up incidence rate over the subgroup combinations that were composed correspondingly to the adjustment for inflammation and OSA, respectively (*P* < 0.01 for each of the subgroups of low and high inflammation and non-OSA; *P* < 0.05 for the subgroup of OSA).

### 3.3 Odd ratios for MetS according to the concurrent presence of high hsCRP and OSA

Table 2 presents odds ratios estimated for the likelihood of MetS according to hsCRP level among non-OSA and OSA participants, respectively. From the univariate analysis without adjustment, odds ratios for MetS among OSA participants with low and high hsCRP levels were 2.49 (95% CI, 1.86–3.34; *P* < 0.001) and 4.04 (95% CI, 2.79–5.85; *P* < 0.001), respectively, with reference to non-OSA participants with low hsCRP levels. From the multivariate analysis with adjustment for age, sex, smoking, alcohol status, and BMI, OSA participants with high hsCRP level had a 1.58-fold increase (95% CI, 1.03–2.40; *P* < 0.05) in the risk of MetS, as compared with non-OSA participants with low hsCRP level. Table 3 shows adjusted odds ratios estimated for the risk of MetS to develop at the 6-year follow-up among the 4 groups of participants who did not have MetS at baseline. With adjusting for age, sex, smoking, alcohol status, BMI, and BMI change at follow-up (*Δ*BMI), OSA participants having higher hsCRP level had a significantly higher risk to develop MetS at the follow-up (OR, 2.22; 95% CI, 1.22–4.03; *P* < 0.05), as compared with non-OSA participants having low hsCRP level.

**Table 3:**
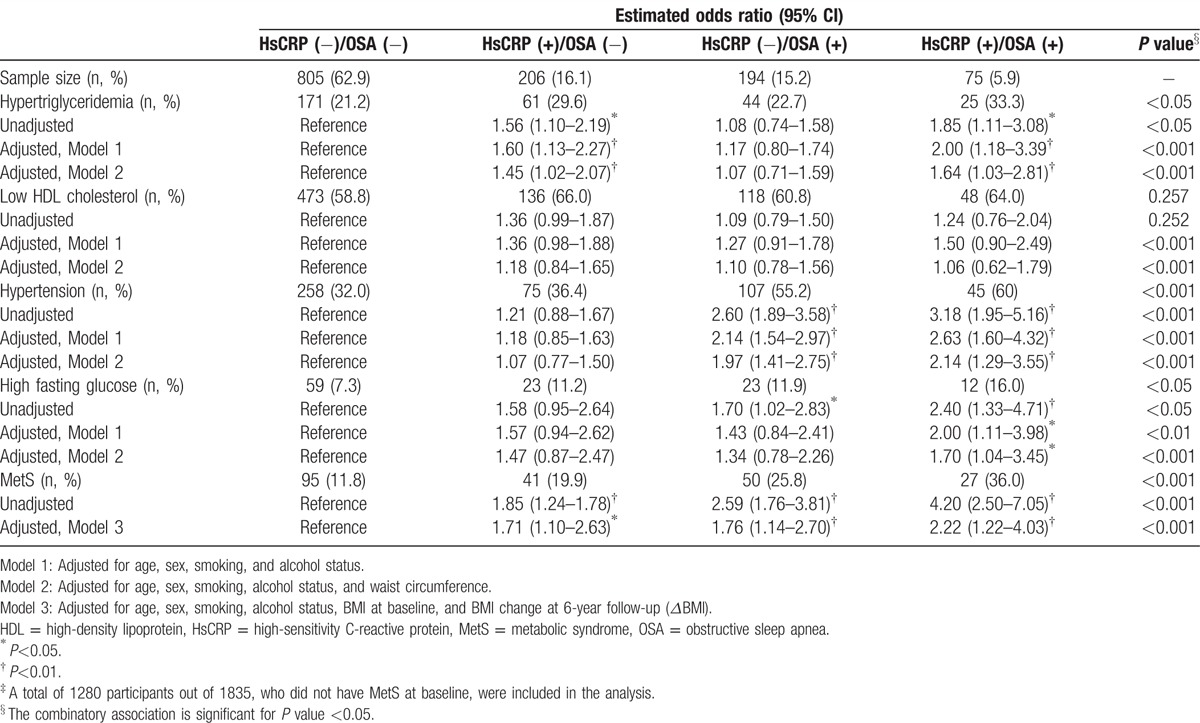
Estimated odds ratios for the development of MetS according to the combinatory presence of inflammation and OSA at 6-year follow-up^‡^.

## 4 Discussion

In a large population-based cohort study, we found that MetS was prevalent among the participants with OSA, particularly in those with high hsCRP level, which manifests an inflammatory phenotype. Even with adjusting for obesity, the prevalence of MetS was significantly higher among OSA participants with a concurrent presence of inflammation, as compared with the corresponding controls (Fig. 1A). The baseline prevalence of MetS in the presence of inflammation only was higher in the subgroup of OSA than in the subgroup of non-OSA, regardless of obesity or nonobesity (Fig. 1C). The incidence of MetS among OSA participants with inflammation was also significantly different in the incidence at the 6-year follow-up (Fig. 2). In addition, we found that there was an important interactive association not only between MetS and OSA presence in those with low hsCRP, but also between MetS and hsCRP in those without OSA (Fig. 1B and C). Even with adjusting for potential confounding factors in logistic regression, OSA participants with low and high inflammation levels had a 1.45- and 1.58-fold risk of MetS at baseline, and 1.76- and 2.22-fold increased risk to develop MetS at the 6-year follow-up, respectively, as compared with the corresponding non-OSA participants with a low inflammatory phenotype, indicating that activation or propagation of inflammatory pathways in OSA could play an important role in accelerating the risk of MetS. To our knowledge, this study is the first to show the combined concurrent effect of OSA and inflammation on the development of MetS in a large population-based study. However, the magnitude and significance of the study findings require replications in other cohort studies.

Considering the ever-increasing body of evidence regarding OSA, it is clear that OSA should be viewed as a chronic systemic inflammatory disease in children as well as in adults.^[11,17]^ Although the exact mechanisms linking OSA to the inflammatory cascade are not clear, intermittent hypoxia and reoxygenation characterized in OSA contribute to cumulative burden of oxidative stress and generation of reactive oxygen species and trigger inflammatory cytokines, thereby promoting insulin resistance and metabolic dysfunction.^[7,32]^ HsCRP is a robust biomarker of underlying systemic inflammation and appears to be an important marker in both cardiovascular disease and metabolic syndrome.^[3,33]^ It is not surprising that increased levels of hsCRP have been reported in both adults^[34–36]^ and children with OSA,^[37,38]^ and that such levels decreased after OSA treatment.^[3,11,39,40]^ However, we should also emphasize that not all studies in adults ^[41,42]^ or children ^[13,14]^ have confirmed this putative association between hsCRP level and OSA, suggesting that the causal relationship between CRP elevation and OSA might not always be phenotypically expressed. As seen in our study (Figs. 1A and 2A), it is likely that the interaction between the severity of OSA and obesity, as well as genetically determined variance, may account for these discrepancies.^[3,17,19]^ This result was also confirmed at the 6-year follow-up for the incidence of MetS, and adjusted odds ratios using multivariate regression analyses. At baseline, the prevalence of MetS was significantly associated with the concurrent presence of inflammation and obesity as compared with those without inflammation and OSA; however, the odds ratios of baseline prevalence were rather close to each other around 1.5 to 1.6 (Table 2). On the other hand, odds ratio for the incidence at 6-year follow-up increased around 1.7 for the groups with either inflammation or OSA, but not both and 2.2 for the group with both inflammation and OSA, which is greater than the odds ratio for the corresponding group of prevalence at baseline, that is, around 1.5 and 1.6, respectively (Table 2). Thus, it is expected that the influence of concurrent presence of inflammation and OSA may appear in time such at least 6 years long as in our study. Additionally, we examined the risk of MetS not only at baseline but also at 6-year follow-up, respectively, in the subgroups of hsCRP (Figs. 1B and 2B) and in the subgroups of OSA (Figs. 1C and 2C). There was an increasing trend over the combination groups accordingly to the controlled subgroups, in the prevalence and incidence of MetS, as seen in the figures. The present study focused on identifying the impact of concurrent presence of inflammation and OSA on MetS with adjusting for obesity, and did not statistically calculate a linear increment in the increasing trend over the combination groups, which is neither necessary nor important of interest.

While the pathophysiologic mechanisms by which combined OSA and inflammation might accelerate glucose tolerance and insulin resistance are not clearly understood, a previous study reported that high CRP level was independently associated with increased risk of MetS.^[33,43]^ The prospective design of the available studies provides a compelling evidence for a causal association between low-grade systemic inflammation and metabolic dysfunction.^[33]^ Moreover, clinical studies show that both TNF-α and IL-6 are higher among patients with OSA compared with BMI-matched controls, respectively, suggesting that these cytokines have a role in insulin resistance.^[44]^ The TNF-α polymorphism, TNF-α (−308A), which is associated with OSA, suggests that genetic variances associated with inflammation account for the differences in inflammatory phenotypes in OSA.^[45,46]^ Recently, an epigenetic study also revealed that the percentage of methylation in the promoter region of the FOXP3 gene is significantly associated with the determinants of inflammatory phenotype among OSA children, supporting the theory that epigenetic modifications play a crucial role in activation of the inflammatory cascade in OSA.^[19]^ Thus, the inflammatory effects of OSA might predispose individuals to synergistically altered metabolic function. A more definitive study should be performed to confirm whether the combination of inflammation and OSA synergistically or additively affects insulin resistance and metabolic dysfunction in experimental and clinical settings.

Over the past several decades, despite substantial evidence from both epidemiological and clinical studies suggesting that OSA is related to metabolic impairment and that there is an independent relationship between OSA and MetS, the issue remains controversial. Most of the published studies were cross-sectional and burdened with methodological limitations, such as small sample sizes, subjective measurement of OSA, and inadequate consideration of confounding factors. It has been reported that the prevalence of MetS among OSA patients varied between 11% and 87% when diagnosed using NCEP-III criteria.^[47–51]^ To date, only a few prospective studies have shown a causal association by demonstrating a higher incidence of diabetes among subjects with OSA,^[50,52]^ however, results have been conflicting. In a longitudinal study of the Wisconsin Sleep Cohort, the risk of developing diabetes over a 4-year period was not significantly different between subjects with an AHI≥15 and those with an AHI <5 after adjusting for age, sex, and body habitus.^[32]^ In contrast, Marshall et al^[53]^, in an Australian population-based cohort study, reported that moderate to severe sleep apnea (15≥RDI) was significantly associated with incidence of diabetes after adjustment for confounding factors. Additionally, data from a community-based sample demonstrated that sleep apnea was a significant predictor of insulin resistance, using the insulin sensitivity index and HOMA-IR at 11 years follow-up.^[52]^ In the present study, we found that the highest prevalence and incidence of MetS was shown among OSA participants with a high inflammatory phenotype, independent of obesity. However, the incidence rate of MetS among individuals with OSA with high hsCRP level relative to those with OSA with low hsCRP level might be affected by the high rate of nonrespondents among OSA subjects with high hsCRP level at follow-up (nonrespondents, n = 212, HsCRP[−]/OSA[−] vs HsCRP[+]/OSA[−] vs HsCRP[−]/OSA[+] vs HsCRP[+]/OSA[+], 10.3% vs 14.5% vs 11.7% vs 13.7%, *P* = 0.184). Another possible explanation for this finding might be the relatively small sample size of OSA subjects with high hsCRP level (n = 75) compared with the other corresponding groups. Thus, confirmatory studies are needed to further define the significance of the combined effect of OSA and subclinical inflammation on development of MetS in a large population-based prospective study.

Compared with previous studies, many of which had methodological limitations, the current study has several strengths. We used a prospective population-based sample and differentiated the effects of inflammation and OSA. In the present study, we randomly selected 1835 subjects from the KoGES cohort. The general characteristics and biochemical data, including BMI and hsCRP levels, between participants and nonparticipants at baseline were not significantly different (*P*>0.05). Thus, our result could be representative of the general population. Another advantage of the present study was the evaluation of OSA using a portable PSG at home, which provides a more realistic assessment of OSA severity than hospital-based studies owing to the maintenance of regular daily habits of sleep, physical activity, and diet in the general population.

Although the present study included a large general population sample, prospective design, and follow-up measurements, several limitations should be acknowledged. First, the evaluation of inflammatory phenotype using only hsCRP level among those with OSA may not sufficiently represent the inflammatory state of participants, as previous studies have suggested that other predictable inflammatory markers and various cytokines are associated with OSA. However, an accumulating body of evidence shows that hsCRP is a reliable and predictable inflammatory maker of various OSA-related comorbidities and subsequent outcomes.^[8]^ Thus, studies should follow with using different OSA-related inflammatory markers such as TNF-alpha and IL-6 to determine whether variances in inflammatory phenotypes among those with OSA may accelerate the development of MetS. Second, our PSG study was performed over 2.5 years. Even though a change of BMI (*Δ*BMI), which is the most crucial predictor of OSA progression, varied little during the follow-up period in the present study, we still believe this time span is short enough not to produce any significant change in OSA severity.^[54,55]^ Accordingly, the effect of weight gain on the incidence of MetS among OSA patients may need to be validated in other study cohorts. Third, although inflammatory conditions can be influenced by participant's nutritional habit and exercise status, this was not considered in the present study. However, these factors might not have affected the inflammatory conditions of participants, as our data showed a difference in hsCRP levels among the 4 groups at the follow-up. Fourth, genetic variances and epigenetic modifications that play an important role in the regulation of an inflammatory phenotype among those with OSA were not examined in the present study. Accordingly, future longitudinal studies that focus on interactions between inflammation-related genetic variances or epigenetic alterations and the development of MetS among those with OSA should be conducted. Finally, we did not elucidate a possibility of reverse causality, whether CPAP treatment or anti-inflammatory therapy for OSA could reduce the risk of MetS. Well-designed, randomized control trials may be needed to address this issue in future research efforts.

In summary, MetS is more prevalent in the concurrent presence of inflammation and OSA, and their combinatory presences may constitute an important determinant of the development of MetS. Additional research is needed to help further determine the significance of the combined effect of OSA and subclinical inflammation on the development of MetS in the context of a reduction in CVD risk.

## Acknowledgments

The authors thank all the participants and research staff of the Institute of Human Genomic Study at Ansan Hospital of the Korea University.
